# Differential Amino Acid, Carbohydrate and Lipid Metabolism Perpetuations Involved in a Subtype of Rheumatoid Arthritis with Chinese Medicine Cold Pattern

**DOI:** 10.3390/ijms17101757

**Published:** 2016-10-21

**Authors:** Hongtao Guo, Xuyan Niu, Yan Gu, Cheng Lu, Cheng Xiao, Kevin Yue, Ge Zhang, Xiaohua Pan, Miao Jiang, Yong Tan, Hongwei Kong, Zhenli Liu, Guowang Xu, Aiping Lu

**Affiliations:** 1 Department of Rheumatology, First Affiliated Hospital of Henan University of TCM, Zhengzhou 450000, China; guoht2009@126.com; 2 Institute of Basic Research in Clinical Medicine, China Academy of Chinese Medical Sciences, Beijing 100700, China; niuxuyantcm@163.com (X.N.); lv_cheng0816@163.com (C.L.); miao_jm@vip.126.com (M.J.); tcmtanyong@126.com (Y.T.); 3 CAS Key Laboratory of Separation Science for Analytical Chemistry, Dalian Institute of Chemical Physics, Chinese Academy of Sciences, Dalian 116023, China; q13581555831@163.com (Y.G.); konghw@dicp.ac.cn (H.K.); 4 School of Medicine, Shanxi Datong University, Datong 037009, China; 5 Institute for Advancing Translational Medicine in Bone & Joint Diseases, School of Chinese Medicine, Hong Kong Baptist University, Kowloon Tong 00852, Hong Kong, China; kkmyue@hkbu.edu.hk (K.Y.); zhangge@hkbu.edu.hk (G.Z.); 6 Department of Scientific Research Administration, China-Japan Friendship Hospital, Beijing 100029, China; xc2002812@126.com; 7 Department of Rheumatology, People Hospital of Yichun City, Yichun 336000, China; 8 Jinan University & Hong Kong Baptist University Joint Laboratory of Innovative Drug Development, Institute of Biomedicine (Guangzhou), Jinan University, Guangzhou 510632, China; szpxh4141@163.net; 9 Institute of Basic Theory of TCM, China Academy of Chinese Medical Sciences, Beijing 100700, China; zhenli_liu@sina.com; 10 E-Institute of Chinese Traditional Internal Medicine, Shanghai Municipal Education Commission, Shanghai 201203, China

**Keywords:** rheumatoid arthritis, Traditional Chinese Medicine, cold pattern, heat pattern, metabolic pathways

## Abstract

Pattern classification is a key approach in Traditional Chinese Medicine (TCM), and it is used to classify the patients for intervention selection accordingly. TCM cold and heat patterns, two main patterns of rheumatoid arthritis (RA) had been explored with systems biology approaches. Different regulations of apoptosis were found to be involved in cold and heat classification in our previous works. For this study, the metabolic profiling of plasma was explored in RA patients with typical TCM cold or heat patterns by integrating liquid chromatography/mass spectrometry (LC/MS) and gas chromatography/mass spectrometry (GC/MS) platforms in conjunction with the Ingenuity Pathway Analysis (IPA) software. Three main processes of metabolism, including amino acid, carbohydrate and lipid were focused on for function analysis. The results showed that 29 and 19 differential metabolites were found in cold and heat patterns respectively, compared with healthy controls. The perturbation of amino acid metabolism (increased essential amino acids), carbohydrate metabolism (galactose metabolism) and lipid metabolism, were found to be involved in both cold and heat pattern RA. In particular, more metabolic perturbations in protein and collagen breakdown, decreased glycolytic activity and aerobic oxidation, and increased energy utilization associated with RA cold pattern patients. These findings may be useful for obtaining a better understanding of RA pathogenesis and for achieving a better efficacy in RA clinical practice.

## 1. Introduction

Rheumatoid arthritis (RA) is a chronic systemic inflammatory autoimmune disease that affects different parts of the body, inevitably causing multi-organ disorders. Traditional Chinese Medicine (TCM) has been used in China for centuries and has shown efficacy in RA treatment [1]. According to TCM theory, pattern differentiation is one of the most important concepts in the practice of TCM, and the clinical efficacy would increase if right TCM pattern is chosen during the treatment process [2,3]. It consists of a series of diagnostic information including symptoms, tongue appearance and pulse feelings of patients [4]. The differentiation of TCM patterns plays a crucial role in TCM clinical practice [5,6]. However, the information of TCM pattern differentiation based on, such as color of tongue, pulse feelings, which is judged by clinical practitioner with subjective factor in part, and our previous studies showed that more easily understandable symptoms and related signs can be used for TCM pattern differentiation which judged by modern biological techniques [7,8].

Numerous proteomic and genomic studies have provided extensive insight into the foundational pathology of RA with the development of systems biology [9,10,11,12]. In order to find the biological basis of different TCM patterns, our previous studies at the transcriptional and metabolomic level on RA TCM patterns were performed to show the different regulation of apoptosis between RA cold and heat patterns [13,14]. However, more exploration in depth on amino acid, carbohydrate and lipid metabolism related to RA heat and cold pattern is further needed for the full picture of RA cold and heat patterns in term of biomedical perspectives.

Notably, emerging evidence implies that RA susceptibility may be involved in the perturbation of metabolism [15,16]. Evidence for altered metabolism associated with chronic rheumatic diseases is found in the biochemical profiles of biological fluids and tissues that arise from a diverse set of metabolic pathways [17]. The metabolomics approach can provide insight into the entire metabolism process and then identify disparities in the metabolites and related metabolic pathways in the complex regulatory network [18]. Based on an analysis of a series of different sample spectra, metabolomics can be used to identify the pathophysiological state, gene function, drug toxicity and efficacy, and associated biomarkers in organisms [19]. The usefulness of metabolic profiling has been demonstrated in the diagnosis of RA [20]. Recent evidence has also demonstrated that a metabolomics approach can be an effective tool in characterizing the metabolic changes of RA [20,21], therefore maybe in deciphering RA patients with cold and heat TCM patterns in our conjecture.

In this study, RA patients with typical cold or heat pattern were enrolled. An integrated platform of LC-MS and GC-MS were applied for sample analysis in this study. Utilizing liquid chromatography/mass spectrometry (LC/MS) and gas chromatography/mass spectrometry (GC/MS) platforms, we investigated the metabolic changes in plasma of RA patients with cold pattern compared with healthy controls and changes in RA patients with heat pattern compared with healthy controls. We conducted the analysis of bio-functions and canonical pathways using the Ingenuity Pathway Analysis (IPA) software (Ingenuity, Redwood City, CA, USA) for the differential metabolites to gain insight into the typical metabolic perturbations network associated with the given patterns of RA.

## 2. Results

### 2.1. Differential Metabolites and Canonical Pathways Involved in Rheumatoid Arthritis (RA) Cold and Heat Patterns

Twenty-nine differential metabolites, including eleven up-regulated metabolites and eighteen down-regulated metabolites (Table S1), were confirmed in the cold pattern of RA patients compared with healthy controls. Nineteen differential metabolites (8 up-regulations and 11 down-regulations) were identified in the heat pattern RA patients compared with healthy controls (Table S2).

To analyze the perturbation involved in different metabolic processes, the canonical pathways of differential metabolites in cold and heat patterns were further explored independently for differences in three main types of metabolic processes: amino acid metabolism, carbohydrate metabolism and lipid metabolism. The related metabolic pathways that are affected by cold pattern are amino acid metabolism (Figure S1A), carbohydrate metabolism (Figure S1B), and lipid metabolism (Figure S1C) and number eight, eight and nine, respectively. As for the heat pattern, the number of related metabolic pathways is six, four and two, respectively (shown in Figure S2).

### 2.2. Comparison Analysis between Cold and Heat Patterns in Amino Acids Metabolism

The “Comparison Analysis” module in IPA was used to elucidate the three main related metabolic processes in detail to compare the shared and different characteristics in metabolic canonical pathways between cold and heat pattern RA.

There were five common pathways between cold and heat patterns (Table 1): aminoacyl-tRNA biosynthesis; valine, leucine and isoleucine biosynthesis; alanine and aspartate metabolism; valine, leucine and isoleucine degradation; glycine, serine and threonine metabolism. Additionally, three unique pathways of cold pattern were identified. l-proline and urea were down-regulated in cold pattern and are associated with the urea cycle and metabolism of amino groups, arginine and proline metabolism. Citric acid, which is related to the glutamate metabolism pathway and participates in the alanine and aspartate metabolic pathways, was also down-regulated in cold pattern.

### 2.3. Comparison Analysis between Cold and Heat Patterns in Carbohydrates Metabolism

There were four shared pathways between cold and heat patterns (Table 2): *glyoxylate* and *dicarboxylate metabolism*, *galactose metabolism*, *inositol metabolism* and *propanoate metabolism*. There were also unique four metabolic pathways specific to cold pattern: *citrate cycle*, *pentose phosphate pathway*, *glycolysis/gluconeogenesis*, and *starch* and *sucrose metabolism*. d-glucose and citric acid, which are participants in the four unique pathways, were down-regulated in cold pattern.

### 2.4. Comparison Analysis between Cold and Heat Patterns in Lipid Metabolism

Regarding lipid metabolism pathways (Table 3), two shared lipid metabolic pathways were found between cold and heat patterns: *glycerolipid metabolism* and *inositol phosphate metabolism*. The metabolites d-glyceric acid and myo-inositol, which are involved in the two aforementioned shared lipid metabolic pathways, were shared in these two groups. Interestingly, the remaining seven pathways were only involved in cold pattern: *fatty acid biosynthesis*, *fatty acid elongation in mitochondria*, *biosynthesis of steroids*, *C21-steroid hormone metabolism*, *linoleic acid metabolism*, *bile acid metabolism*, and *fatty acid metabolism*.

### 2.5. Integrative Analysis in Amino Acids, Carbohydrates and Lipid Metabolism between Cold and Heat Patterns

**Figure 1:**
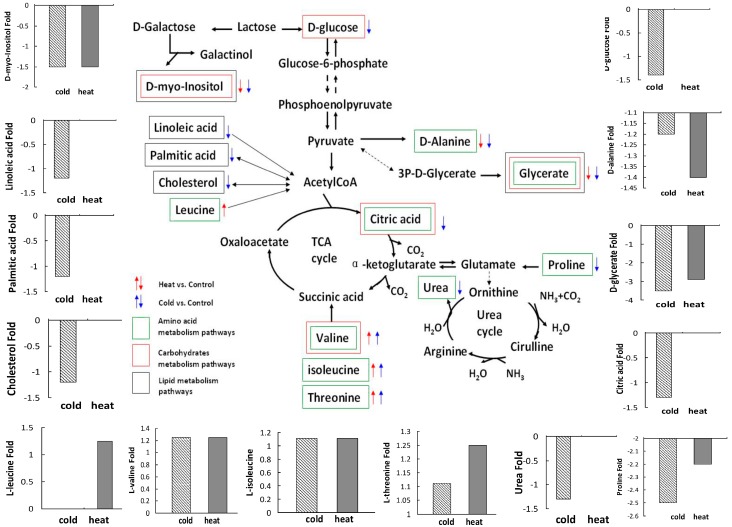
An overview of metabolic imbalances in rheumatoid arthritis (RA) patients with cold and heat patterns. A schematic representation of amino acid, carbohydrate and lipid metabolism in the center, showing representative metabolite changes connected to the cold pattern and heat pattern of RA patients. The **direction of the arrows** represents the regulation direction of metabolites; the **red arrows** represent metabolites in heat pattern RA compared to healthy controls; the **blue arrows** represent metabolites in cold pattern RA compared to healthy controls. The **textboxes outside the molecules** represent different types of metabolic pathways: **green** represents amino acid metabolism; **red** represents carbohydrate metabolism; and **black** represents lipid metabolism.

Based on the 29 and 19 differential metabolite alterations and the related perturbations of various biological pathways in RA patients with TCM cold or heat pattern, the metabolic processes were disturbed in both RA cold and heat patterns in certain ways. There were many common metabolic pathway perturbations in amino acid, carbohydrate and lipid metabolism between cold and heat patterns (Table 1, Table 2 and Table 3). Some common metabolites with same change trend involved in the perturbation of amino acid metabolism, carbohydrate metabolism and lipid metabolism in the cold and heat patterns group, and interestingly some unique disruptions of metabolic pathways specific to cold pattern RA patients were also found. By integrating such clues, a schematic view of amino acid, carbohydrate and lipid metabolism in RA cold and heat patterns is set up to show the converging and diverging metabolic characteristics between the cold and heat patterns of RA (Figure 1).

## 3. Discussion

TCM takes a systemic view of the diagnosis and treatment of disease. The use of metabolomics coincides with this holistic thinking, suggesting the possibility of employing metabolomics studies to elucidate the basis of TCM [22]. Pattern classification plays a central role in the diagnosis and therapy of RA in TCM clinical practice. In TCM clinical practice, the symptoms of RA can be classified into different patterns: the cold, heat and deficiency patterns are key patterns in RA. These patterns can be identified individually or concomitantly, such as deficiency-cold, deficiency-hot, or intermingled cold and hot patterns. For TCM deficiency pattern identification, deformity and inhibited bending and stretching in limbs are key manifestations, and generally these symptoms clinically occur in the later stage of disease or later than other common articular symptoms such as pain with cold or heat feeling and swelling. Thus, in most cases, deficiency pattern is believed as result of prolonged disease or delayed treatment. Therefore, in our study, the cold and heat patterns, which can be screening at early stage of disease, was chosen as our main study pattern.

Our previous clinical study [2] suggested that the effective rate of the biomedical combination therapy shows different results in RA cold pattern group and RA heat pattern group. It made it reasonable to make the hypothesis that the heterogeneity of pattern in TCM is subsistent and this pattern classification may have its own specific biomarkers. Numerous metabolomics approaches have gained great prominence in investigating systematic metabolic characteristics of RA; Metabolic signaling programmers are integral to T cell activation, differentiation and function [23], which implied the closely association between metabolism and immunity. Our other study [14] proved an increased urea production in RA heat pattern, indicating more protein breakdown than in RA cold pattern. In addition, Proline and oxo-Proline are increased in RA heat pattern, which is abundantly released during collagen breakdown. Additionally, in RA cold pattern Leucine levels are raised. It can indicate protein synthesis, which is in agreement with normal urea levels found in RA cold pattern. Even so, the metabolic characteristics of a given TCM pattern of RA, specifically the primary cold or heat pattern, which are the most common patterns of RA in clinical TCM, remain largely unknown. In this study, 29 and 19 differential metabolite alterations were found in RA cold and heat patterns, respectively. The results revealed the perturbations of various biological pathways in RA patients with TCM cold or heat pattern. Interestingly, some unique disruptions of metabolic pathways specific to cold pattern RA patients were shown. It suggests the possibility of distinguishing cold and heat patterns RA at the metabolic level (Figure 1).

For amino acid metabolism, we found that some amino acids were up-regulated in both cold and heat patterns, including valine, isoleucine and threonine. This finding is consistent with similar RA studies from our group [24]. All of the shared amino acids in cold and heat patterns were essential amino acids (EAA), amino acids that are not produced by human body and only supplied by external food. The increased EAA in plasma suggested that the utilization of these amino acids was decreased during amino acid metabolism in both the cold and heat patterns of RA. In addition, d-alanine was down-regulated in both cold and heat patterns. The literature implies that a decrease in alanine concentration may be attributed to the degradation of the collagen framework and to the eventual loss of degradation products that are transported from cartilage into the synovial cavity [25]. For unique differential metabolites of cold pattern compared with healthy controls, up-regulated urea, l-proline and citric acid represented three characteristic pathways of cold pattern. Citric acid disturbance can be found in animal models of RA [26], and up-regulated l-proline and urea indicate more protein and collagen breakdown in cold pattern. Other work [27] also shows that the cold pattern patients experience low level of energy metabolism, which leptin was confirmed as a biomarker in cold pattern.

Five identified metabolites participate in carbohydrate metabolic pathways. Citric acid, d-glyceric acid, myo-inositol, d-glucose and l-valine were found in both cold pattern and heat pattern RA. Among these metabolites, d-glyceric acid and myo-inositol were decreased, and l-valine was increased. These three metabolites are involved in four common pathways in the two groups: glyoxylate and dicarboxylate metabolism, galactose metabolism, inositol metabolism and propanoate metabolism. Galactose metabolism might be related to chronic inflammation [28], and the presence of galactose is important for stabilizing the structure of the entire branched carbohydrate chain. Its absence correlates with the severity of RA [29]. Thus, the perturbation of galactose metabolism might be an important characteristic of cold and heat pattern RA. Citric acid and d-glucose, the unique identified metabolites in cold pattern, are involved in special pathways: the citrate cycle, the pentose phosphate pathway, starch and sucrose metabolism, and glycolysis/gluconeogenesis. Some studies have reported that glucose metabolism plays an important role in the pathogenesis of RA and that there is increased glycolytic activity in the synovial tissue of RA patients [30]. In our study, we observed a decrease in the d-glucose and citric acid concentrations in the plasma of cold pattern RA patients, indicating decreased glycolytic activity and aerobic oxidation. These decreases seem to be another unique characteristic of cold pattern RA patients. If more verification can be performed for these metabolic characteristics, it can be regarded as potential symbols for distinguishing between RA cold and heat patterns.

One point to note is that there were dramatic differences in the lipid metabolic pathways between cold pattern and heat pattern. The identified metabolites related to heat pattern participate in two lipid metabolic pathways: glycerolipid metabolism and inositol phosphate metabolism. In contrast, most of the lipid canonical metabolic pathways were related to the cold pattern. Palmitic acid, cholesterol and linoleic acid were down-regulated only in the cold pattern. The down-regulated palmitic acid, cholesterol and linoleic acid signified an increase of their utilization as energy sources in the citrate cycle. We considered this to be the specific metabolic character of cold pattern RA. It has been reported that an adverse lipid profile or dyslipidemia is an important risk factor for cardiovascular disease (CVD) in the general population [31,32]. The evidence for increased cardiovascular risk in patients with RA is convincing [33,34]. Some researchers have established the disorder of lipid metabolism in RA patients as having anti-lipoprotein antibodies that may be connected to dyslipoproteinemia [35]. Therefore, we speculate that because of the markedly decreased lipid metabolism, cold pattern RA patients may have a higher risk of CVD compared with heat pattern RA patients. This finding correlates well with our previous work [13].

The conclusions of this study must be weighed within the confines of some limitations of this data source. One limitation is the complexity of TCM pattern. Here we choose two types of patterns (cold and heat), though they are typical and important, there are more patterns, such as deficiency pattern and some combinations of patterns. Another limitation is the relatively small sample size. Though we collected more samples than our previous studies [2] and applied more advanced techniques in the testing approaches, more samples could be further needed for deep exploration on the metabonomic profilings of TCM pattern of RA. Other than that, the patient samples were tested only at one time point reduce the reliability of the conclusion, a follow-up study to verify the patterns reproducible could be also needed in the future.

Moreover, our study found some common metabolites with same change trend involved in the perturbation of amino acid metabolism, carbohydrate metabolism and lipid metabolism in cold and heat pattern group. We thought this issue is not contradictory. The cold and heat patterns belong to the disease of rheumatoid arthritis; these common metabolites may be characteristic symbols of the RA itself. Our results show that there are still some differences in the variation degree among these common metabolites between cold and heat patterns. These common metabolites between cold and heat pattern might be the potential study focus for an RA cold and heat pattern sstudy.

Taken together, this reveals the underlying perturbations of amino acid, carbohydrate, and lipid metabolism of RA with TCM cold and heat pattern, including the perturbation of amino acid metabolism (increased essential amino acids), carbohydrate metabolism (galactose metabolism) and lipid metabolism, which are involved in both cold and heat patterns RA, and more metabolic perturbation in protein and collagen breakdown, decreased glycolytic activity and aerobic oxidation, and increased energy utilization related to RA with cold pattern. These characteristics could be used to distinguish cold pattern RA from heat pattern RA, and these results could provide useful clues on the stratification of RA patients for choosing an effective therapeutic strategy for these patients.

## 4. Materials and Methods

### 4.1. Chemicals

HPLC-grade acetonitrile was supplied by Merck (Darmstadt, Germany). Water was produced by the Milli-Q ultra-pure water system (Millipore, Billerica, MA, USA). Both methanol and formic acid (HPLC grade) were purchased from Tedia (Fairfield, OH, USA). Methoxyamine hydrochloride, *N*-methyl-*N*-(trimethylsilyl) trifluoroacetamide (MSTFA) and pyridine were purchased from Sigma-Aldrich (St. Louis, MO, USA). Metabolite standards were also obtained from Sigma-Aldrich.

### 4.2. Patients and Sample Preparation

All experiments were performed in accordance with relevant guidelines and regulations from China Academy of Chinese Medical Sciences, and all protocols involving human subjects were approved by the ethics committee of China-Japan Friendship Hospital and China Academy of Chinese Medical Sciences. Due to the existence of different pattern complexity and conversion, typical and pure RA cold or heat patterns are not often present. Different from the patient recruit process in our previous studies, patients with typical TCM cold and heat patterns were confirmed under a rigorous selection process from a patient group with big size. Firstly, 150 female patients who met the American College of Rheumatology criteria for RA were recruited from China-Japan Friendship Hospital for TCM cold and heat pattern screening. Patients were required to meet the following enrollment criteria: Adults ≥18 years of age with a diagnosis of RA, a disease duration <3 years. Among them, 57 RA patients (Table 4) met the typical TCM cold and heat pattern diagnostic criteria based on our previous study [13,14]. Briefly, a questionnaire was designed to record the pattern information of the RA patients based on traditional Chinese medicine theory and our previous study experience. The main characteristics of RA cold pattern include the fixed severely pain in a joint or muscle and can be alleviated by heat. Loose stools, clear profuse, thin white tongue coating and tight pulse were often accompanied with RA cold pattern patients. As for RA heat pattern, these patients are characterized by severe pain with hot, red, swollen and inflamed joints. Cold compress can relieve the pain feeling. Fever, thirst, red face, irritability, restlessness, constipation and dark urine are often presented at these patients. Medication history includes continuously received non-steroidal anti-inflammatory drugs, DMARDs, biological agents or corticosteroids for over 6 months, as well as the aforementioned medicines within one month were excluded from this study. Patients with other serious organ disorder and pregnant women were also excluded from the study. Healthy controls with normal physical examination results and non-arthritis disorder history were recruited in China-Japan Friendship Hospital.

Of these 57 patients, 28 RA patients were categorized in the cold pattern group, and 29 RA patients were categorized in the heat pattern group. Twenty-three healthy female volunteers (40.6 ± 6.1 years old) participated in this study. We designed the healthy group as third-party reference for comparison between cold and heat patterns to reduce the possible confounding factors. Blood samples were collected from all RA patients and healthy control subjects in the morning and the obtained plasma were instantly frozen at −80 °C for the next metabolomics analysis [36]. Plasma aliquots (100 μL) were mixed with 300 μL methanol and lyophilized at 10 °C after vortex and centrifuging. All protocols involving human subjects were approved by the ethics committee of China-Japan Friendship Hospital (ethics ID: 2012-62, 7 July 2012), and oral informed consent forms were agreed to by all participants before commencing the study.

### 4.3. LC/MS and GC/MS Analysis

The LC/MS and GC/MS analyses were performed according to previous instructions [37]. Briefly, chromatographic separation was performed on Waters ACQUITY UPLC system (Waters Corp., Milford, CT, USA), equipped with an auto sampler. UPLC separation was carried on an Acquity UPLC™ BEH 2.1 mm (i.d.) × 100 mm C_8_ column with a 1.7 μm particle size (Waters Corp., Milford, CT, USA). The mobile phase was composed of acetonitrile and water, containing 0.1% formic acid, 0.1% formic acid in water (A) and acetonitrile (B). The gradient program was 5% B for 0.5 min, changed to 65% B linearly within 13.5 min, increased to 100% B during the next 11.5 min, then held for 2.5 min, and finally decreased back to 5% B and equilibrated for 2 min. The flow rate was 0.35 mL/min, and the injection volume was 3 μL. The column temperature was set to 35 °C. A Micromass Q-TOF MS system (Waters Corp., Manchester, UK), equipped with an electrospray ionization (ESI) source was used for simultaneous determination. The positive ESI conditions were as follow: capillary voltage, 3100 V; cone voltage, 30 V; collision energy, 4 eV; desolvation gas, 500 L/h; cone gas, 50 L/h; desolvation temperature, 300 °C; source temperature, 120 °C. The mass spectrometric data were collected in centroid mode from *m*/*z* 100 to 1000 with a scan time of 0.1 s. Leucine–enkephalin was used as the lock mass. The collision energy was set at 20 eV.

GC/MS analysis was performed on an Agilent 7890/5975C-GC/MSD system equipped with a 30 m × 0.25 mm (i.d.) fused-silica capillary column chemically bonded with 0.25 μm HP-5 stationary phase (J & W Scientific, Folsom, CA, USA). Helium was used as the carrier gas at a flow rate of 1.0 mL/min. Oven temperature was kept at 70 °C for 2 min, varied from 70 °C to 220 °C at 3 °C/min, from 220 °C to 270 °C at 5 °C /min, from 270 °C to 320 °C (5 min held) at 10 °C /min. The injector and interface temperatures were held at 300 °C and 230 °C, respectively. The detector voltage was set at 1.38 kV, and the solvent delay time was set at 8.5 min. The scanning time was 3.1 s over range of 33–500 amu. The sample of 1 μL was injected in the split mode injection (split ratio, 10:1). For GC-MS analysis, 65 μL of methoxyamine hydrochloride (20 mg/mL in pyridine) was added to oximate and incubated at 40 °C for 90 min. Then, 65 μL of MSTFA was pipetted into the solution for trimethylsilylation at 40 °C for 60 min.

### 4.4. Data Analysis

The Micromass MarkerLynx Applications Manager version 4.0 (Waters Ltd., Manchester, UK) and home-made software were used for raw LC/MS and GC/MS data processing, respectively. The normalization of each variable was performed at the base of the sum of ion intensities in the LC/MS and GC/MS dataset. A total of 313 and 90 variables were generated from the LC/MS data and GC/MS data, respectively. The differential metabolites in cold and heat patterns compared with healthy controls were confirmed by Student’s *t*-test with *p* < 0.05. Chen’s approach [38] and NIST database (NIST08, NIST, USA) were used for metabolite identification in LC/MS and GC/MS date, respectively. Compounds with a higher degree similarity (>75%) were validated by available commercial standard.

### 4.5. Pathway Analysis Using Ingenuity Pathways Analysis Software

Ingenuity Pathways Analysis (IPA, Ingenuity^®^ Systems, http://www.ingenuity.com, Redwood City, CA, USA) is a software application that enables biologists and bioinformaticians to identify the biological mechanisms, pathways and functions that are most relevant to their experimental datasets, genes or metabolites of interest [39,40,41]. The Human Metabolome Database (HMDB) accession number and metabolite names were used as the identifiers of each metabolite dataset. Two datasets were constructed in this study: the identified differential metabolites of cold pattern compared with healthy controls (cold pattern vs. healthy controls) and the identified differential metabolites of heat pattern compared with healthy controls (heat pattern vs. healthy controls). Each dataset was saved as an Excel file. Each metabolomics dataset with identifier (.xls file) was inputted into the IPA software. We utilized the IPA analysis system with the “Metabolomics analysis” module to analyze the identified candidate metabolites. The proof-of-knowledge based IPA was performed to characterize the biomarker metabolites confirmed by the pattern recognition analyses, evaluate the biomarkers based on their metabolic associations in biological function, and build networks of the identified differential metabolites. Canonical pathway analysis of those metabolites was also performed by IPA. Moreover, the common and different points of canonical pathways between cold pattern and heat pattern were determined using the comparison analysis module of IPA. The criteria used to obtain the enriched network stipulated that the score should be higher than 1. Here, the score is -10 logarithms of *Fisher*’s exact test *p*-values in both network and canonical pathway analysis by IPA.

## Figures and Tables

**Table 1:** Comparison of cold pattern vs. healthy control and heat pattern vs. healthy control in amino acids metabolism (↑, upregulation, ↓, downregulation, N.S, no significance).

No.	Pathway	Molecules	Cold Pattern vs. Healthy Control			Heat Pattern vs. Healthy Control		
			Fold	Regulations	*p* Value	Fold	Regulations	*p* Value
1	Aminoacyl-tRNA biosynthesis	l-isoleucine	1.11	↑	5.94 × 10^−7^	1.11	↑	4.21 × 10^−8^
		l-leucine	N.S	N.S		1.25	↑	
		l-threonine	1.11	↑		1.25	↑	
		l-valine	1.25	↑		1.25	↑	
		l-proline	2.5	↓		N.S	N.S	
2	Valine, leucine and isoleucine biosynthesis	l-isoleucine	1.11	↑	1.87 × 10^−4^	1.11	↑	1.54 × 10^−7^
		l-leucine	N.S	N.S		1.25	↑	
		l-valine	1.25	↑		1.25	↑	
3	Urea cycle and metabolism of amino groups	l-proline	2.5	↓	1.31 × 10^−3^	N.S	N.S	N.S
		Urea	1.3	↓		N.S	N.S	N.S
4	Alanine and aspartate metabolism	d-alanine	1.2	↓	1.50 × 10^−3^	1.4	↓	3.21 × 10^−2^
		Citric acid	1.3	↓		N.S	N.S	
5	Valine, leucine and isoleucine degradation	l-isoleucine	1.11	↑	3.26 × 10^−3^	1.11	↑	1.23 × 10^−5^
		l-leucine	N.S	N.S		1.25	↑	
		l-valine	1.25	↑		1.25	↑	
6	Glycine, serine and threonine metabolism	d-glyceric acid	3.5	↓	4.96 × 10^−3^	2.9	↓	1.53 × 10^−3^
		l-threonine	1.11	↑		1.25	↑	
7	Arginine and proline metabolism	l-proline	2.5	↓		N.S	N.S	N.S
		Urea	1.3	↓		N.S	N.S	N.S
8	Glutamate metabolism	Citric acid	1.3	↓	5.91 × 10^−2^	N.S	N.S	N.S

**Table 2:** Comparison of cold pattern vs. healthy control and heat pattern vs. healthy control in carbohydrates metabolism (↑, upregulation, ↓, downregulation, N.S, no significance).

No.	Pathway	Molecules	Cold Pattern vs. Healthy Control			Heat Pattern vs. Healthy Control		
			Fold	Regulations	*p* Value	Fold	Regulations	*p* Value
1	Glyoxylate and dicarboxylate metabolism	Citric acid	1.3	↓	9.38 × 10^−4^	N.S	N.S	N.S
		d-glyceric acid	3.5	↓		2.9	↓	2.55 × 10^−2^
2	Galactose metabolism	myo-inositol	1.5	↓	1.80 × 10^−3^	1.5	↓	3.52 × 10^−2^
		d-glucose	1.4	↓		N.S	N.S	
3	Inositol metabolism	myo-inositol	1.5	↓	1.02 × 10^−2^	1.5	↓	5.77 × 10^−3^
4	Citrate cycle	Citric acid	1.3	↓	4.48 × 10^−2^	N.S	N.S	N.S
5	Pentose phosphate pathway	d-glucose	1.4	↓	4.39 × 10^−2^	N.S	N.S	N.S
6	Propanoate metabolism	l-valine	1.25	↑	7.57 × 10^−2^	1.25	↑	4.33 × 10^−2^
7	Starch and sucrose metabolism	d-glucose	1.4	↓	8.18 × 10^−2^	N.S	N.S	N.S
8	Glycolysis/gluconeogenesis	d-glucose	1.4	↓	9.38 × 10^−2^	N.S	N.S	N.S

**Table 3:** Comparison of cold pattern vs. healthy control and heat pattern vs. healthy control in lipid metabolism (↓, downregulation, N.S, no significance).

No.	Pathway	Molecules	Cold Pattern vs. Healthy Control			Heat Pattern vs. Healthy Control		
			Fold	Regulations	*p* Value	Fold	Regulations	*p* Value
1	Fatty acid biosynthesis	Palmitic acid	1.2	↓	1.76 × 10^−2^	N.S	N.S	N.S
2	Fatty acid elongation in mitochondria	Palmitic acid	1.2	↓	4.12 × 10^−2^	N.S	N.S	N.S
3	Biosynthesis of steroids	cholesterol	1.2	↓	5.2 × 10^−2^	N.S	N.S	N.S
4	C21-steroid hormone metabolism	cholesterol	1.2	↓	5.2 × 10^−2^	N.S	N.S	N.S
5	Linoleic acid metabolism	Linoleic Acid	1.2	↓	6.43 × 10^−2^	N.S	N.S	N.S
6	Bile acid metabolism	Linoleic Acid	1.2	↓	7.4 × 10^−2^	N.S	N.S	N.S
7	Glycerolipid metabolism	d-glyceric acid	3.5	↓	9.8 × 10^−2^	2.9	↓	5.64 × 10^−2^
8	Fatty acid metabolism	Palmitic acid	1.2	↓	1.28 × 10^−1^	N.S	N.S	N.S
9	Inositol phosphate metabolism	myo-inositol	1.5	↓	1.44 × 10^−1^	1.5	↓	8.4 × 10^−2^

**Table 4:** Clinical characteristics baseline of rheumatoid arthritis (RA) patients with cold and heat patterns.

Item	RA with Cold Pattern (*n* = 28)	RA with Heat Pattern (*n* = 29)
Age, mean (S.D.), years	46.7 (9.7)	42.0 (1.7)
RA disease duration, mean (S.D.), years	1.5 (0.9)	1.3 (1.1)
ESR, mean (S.D.), mm/h	20.2 (10.3)	21.5 (9.6)
RF, positive, *n* (%)	14 (50)	16 (55.2)
DAS28-ESR, mean (S.D.)	2.3 (0.6)	2.3 (0.5)
Anti-CCP, positive, *n* (%)	19 (67.9)	21 (72.4)

RA, rheumatoid arthritis; S.D., standard deviation; ESR, erythrocyte sedimentation rate; RF, rheumatoid factor; CCP, cyclic citrullinated peptide.

## Supplementary Materials

Supplementary materials can be found at www.mdpi.com/1422-0067/17/10/1757/s1.
