# Supplementary material for: Differential Amino Acid, Carbohydrate and Lipid Metabolism Perpetuations Involved in a Subtype of Rheumatoid Arthritis with Chinese Medicine Cold Pattern
Source: Int J Mol Sci. 2016 Oct 21;17(10):1757. doi: 10.3390/ijms17101757 (PMC5085781; doi:10.3390/ijms17101757)
Supplement: Supplementary file 1 [file ijms-17-01757-s001.pdf]

Supplementary Materials: Differential Amino Acid, Carbohydrate and Lipid Metabolism Perpetuations Involved in a Subtype of Rheumatoid Arthritis with Chinese Medicine Cold Pattern

Hongtao Guo, Xuyan Niu, Yan Gu, Cheng Lu, Cheng Xiao, Kevin Yue, Ge Zhang, Xiaohua Pan, Miao Jiang, Yong Tan, Hongwei Kong, Zhenli Liu, Guowang Xu and Aiping Lu

Table S1. Identified differential metabolites between rheumatoid arthritis (RA) cold pattern patients and healthy controls.

Metabolite	ID	Fold Change (Cold vs. Control)	False Discovery Rate (FDR) *p* Value
**Up-Regulated**			
Glycochenodeoxycholate	HMDB00637	3.33	0.009
Phophatidylethanolamine (38:6)	HMDB09454	1.43	<0.001
Phophatidylethanolamine (32:1)	HMDB08827	1.43	<0.001
Lysophosphatidylcholine (18:2)	HMDB10386	1.43	<0.001
L-valine	HMDB00883	1.25	0.008
Phosphatidylcholine (34:2)	HMDB07880	1.25	0.026
Phosphatidylcholine (36:4)	HMDB07889	1.25	0.003
Phosphatidylcholine (36:5)	HMDB07891	1.25	0.007
Phosphatidylcholine (34:3)	HMDB07881	1.25	0.007
L-threonine	HMDB00167	1.11	0.041
L-isoleucine	HMDB00172	1.11	0.011
**Down-Regulated**			
Palmitic acid	HMDB00220	0.83	0.001
Cholesterol	HMDB00067	0.83	<0.001
Linoleic acid	HMDB00673	0.83	0.011
D-alanine	HMDB01310	0.83	0.014
Urea	HMDB00294	0.77	0.001
Stearic acid	HMDB00827	0.77	<0.001
Citric acid	HMDB00094	0.77	0.013
Phosphatidylcholine (32:1)	HMDB13404	0.77	0.026
Tetradecanoylcarnitine	HMDB05066	0.77	0.006
Hexadecanoylcarnitine	HMDB06210	0.77	0.002
D-glucose	HMDB00122	0.71	<0.001
Myoinositol	HMDB00211	0.67	<0.001
Dodecanoylcarnitine	HMDB00651	0.67	0.016
Oleic acid	HMDB00207	0.59	<0.001
Carnitine	HMDB00062	0.59	0.018
3-hydroxybutyric acid	HMDB00357	0.50	0.013
L-proline	HMDB00162	0.40	<0.001
D-glyceric acid	HMDB00139	0.29	<0.001

Table S2. Identified differential metabolites between RA heat pattern patients and healthy controls

Metabolite	ID	Fold Change (Heat vs. Control)	False Discovery Rate (FDR) *p* Value
		**Up-Regulated**	
Lysophosphatidylcholine (18:2)	HMDB10386	1.43	<0.001
Phophatidylethanolamine (38:6)	HMDB09454	1.43	<0.001
L-valine	HMDB00883	1.25	0.002
L-threonine	HMDB00167	1.25	0.036
L-leucine	HMDB00687	1.25	0.043
Phosphatidylcholine (34:2)	HMDB07880	1.25	0.042
Phosphatidylcholine (34:3)	HMDB07881	1.25	0.029
L-isoleucine	HMDB00172	1.11	0.038
		**Down-Regulated**	
Phosphatidylcholine (32:0)	HMDB07871	0.83	0.002
Oleic acid	HMDB00207	0.71	0.009
D-alanine	HMDB01310	0.71	0.01
Phosphatidylcholine(34:1)	HMDB07879	0.71	<0.001
Myoinositol	HMDB00211	0.67	<0.001
D-glucose	HMDB06564	0.67	<0.001
Carnitine	HMDB00062	0.63	0.008
Phosphatidylcholine (32:1)	HMDB13404	0.56	<0.001
L-proline	HMDB03411	0.45	<0.001
D-glyceric acid	HMDB00139	0.34	0.001
Phytosphingosine	HMDB04610	0.31	0.029

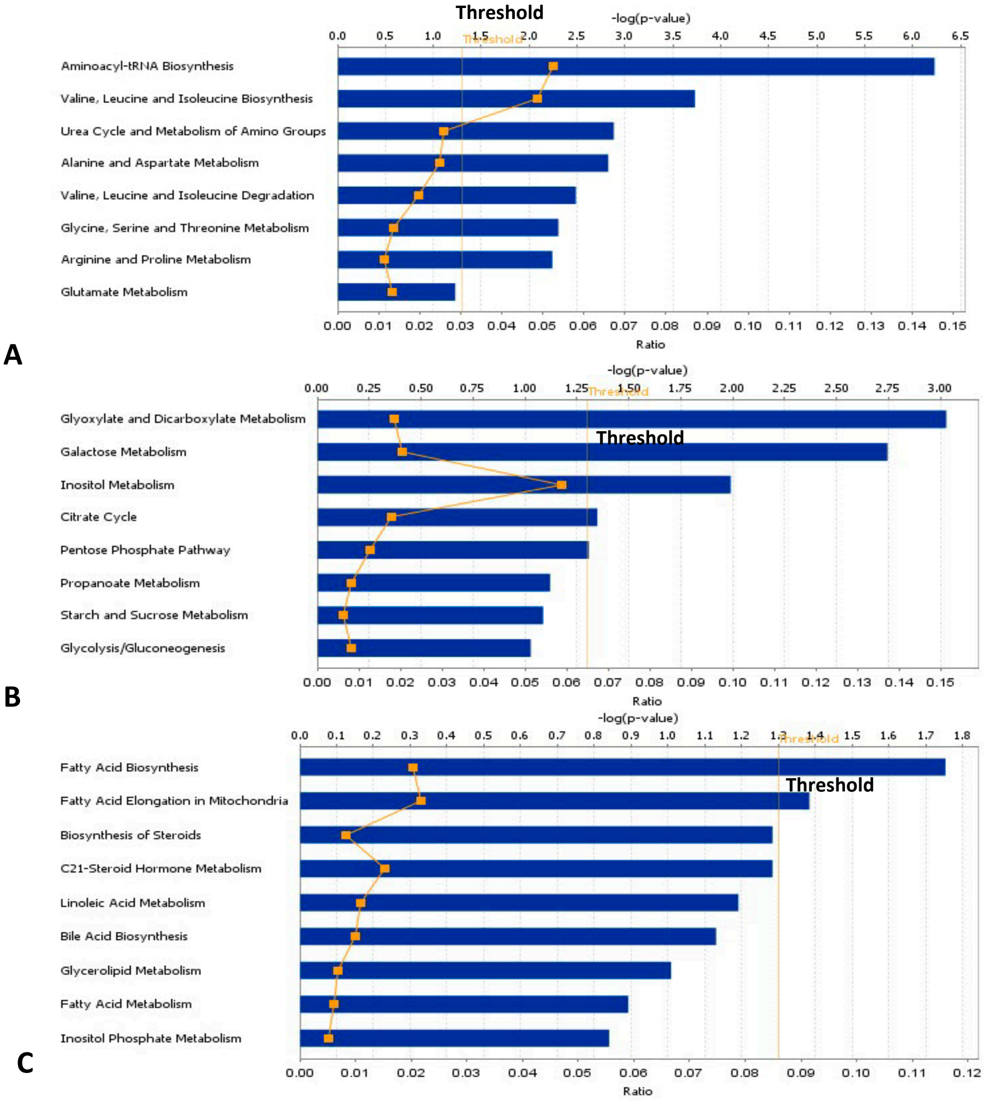

Figure S1. The canonical pathways involved in amino acids, carbohydrates and lipids metabolism between RA cold pattern patients and healthy controls. (**A**) amino acids metabolism related pathway; (**B**) carbohydrates metabolism related pathway; (**C**) lipids metabolism related pathway.

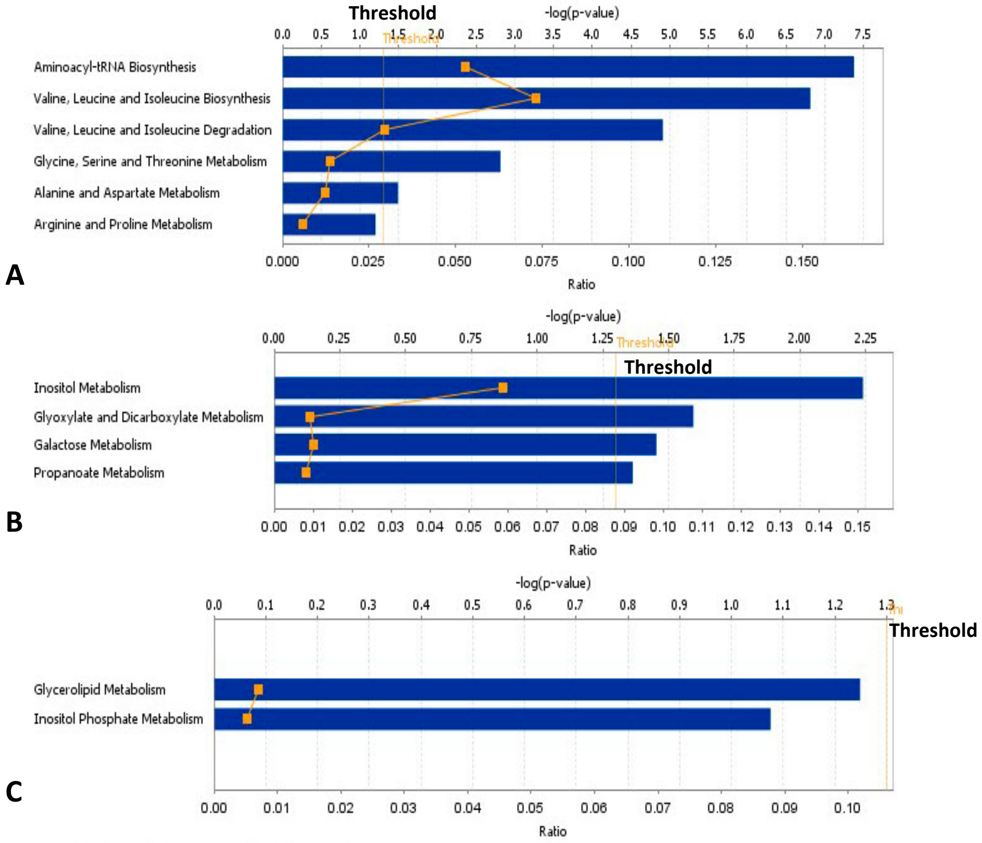

Figure S2. The canonical pathways involved in amino acids, carbohydrates and lipids between RA heat pattern patients and healthy controls. (**A**) amino acids metabolism related pathway; (**B**) carbohydrates metabolism related pathway; (**C**) lipids metabolism related pathway.
